# Band structure engineering of NiS_2_ monolayer by transition metal doping

**DOI:** 10.1038/s41598-021-84967-3

**Published:** 2021-03-11

**Authors:** H. Khalatbari, S. Izadi Vishkayi, M. Oskouian, H. Rahimpour Soleimani

**Affiliations:** 1 Computational Nanophysics Laboratory (CNL), Department of Physics, University of Guilan, P. O. Box 41335-1914, Rasht, Iran; 2 School of Physics, Institute for Research in Fundamental Science (IPM), P. O. Box 19395-5531, Tehran, Iran

**Keywords:** Applied physics, Condensed-matter physics

## Abstract

By using density functional theory calculations, we have studied the effects of V-, Cr-, Mn-, Fe- and Co-doped on the electronic and magnetic properties of the 1T-NiS_2_ monolayer. The results show that pure 1T-NiS_2_ monolayer is a non-magnetic semiconductor. Whereas depending on the species of transition metal atom, the substituted 1T-NiS_2_ monolayer can become a magnetic semiconductor (Mn-doped), half-metal (V- and Fe-doped) and magnetic (Cr-doped) or non-magnetic (Co-doped) metal. The results indicate that the magnetism can be controlled by the doping of 3d transition metal atoms on the monolayer. In this paper, the engineering of the electric and magnetic properties of 1T-NiS_2_ monolayer is revealed. It is clear that it could have a promising application in new nanoelectronic and spintronic devices.

## Introduction

Two-dimensional (2D) graphene, despite having significant physical and chemical properties, has a zero energy gap that limits its applications for use in electronic devices. For this reason, in recent years, new 2D materials with measurable energy gaps such as transitional metal dichalcogenides (TMDs) with the chemical formula MX_2_ (M: transition metal (TM), X: chalcogen (= S, Se and Te)) have attracted a lot of attention due to the wide range of applications in optoelectronics^1^, nanoelectronics^2^, photovoltaics and photodetection^3^. Most 2D TMDs have two stable phases 2H (honeycomb configuration) and 1T (central honeycomb configuration). In other words, 2H and 1T phase can have trigonal prismatic and antiprismatic symmetry, respectively^4–6^. Perhaps in some monolayers, 2H phase is more stable than 1T phase, but this rule does not correct to all monolayers. For example, MoS_2_ monolayer in 2H phase is a semiconductor, however 1T phase for this monolayer is a metal. For this reason, 1T-MoS_2_ monolayer used as a superior supercapacitor electrode material^7^. In addition, some monolayers were synthesized in 2H phase and others in 1T phase. As, MoS_2_^4^, MoSe_2_ and MoTe_2_^8^ were synthesized experimentally in 2H phase and PtS_2_^9^, PtSe_2_^10^ and NiTe_2_^11^ in 1T phase. While the synthesis of 1T phase NiSe_2_^12^ and PtTe_2_^13^ has been performed under laboratory conditions.

Though many pure 2D materials are non-magnetic semiconductors, there are many scientific methods to create magnetization in the non-magnetic 2D systems such as vacancy^14,15^, substitutional doping^16,17^, surface adsorptions^18^ and strain^19^. Based on previous studies, TM doping used as an effective method to create magnetization properties in TMDs. For example, Yang Baishun et al. have investigated the effects of TM atoms, alkali metals and alkaline-earth on the electronic structure and magnetic properties of 1T-ZrS_2_ monolayer by using first-principle calculations^20^. The electronic and magnetic properies of monolayer 1T-ZrSe_2_ in the presence of doping have checked by Xu Zhao and Colleagues^21^. Also, X Wang et al. have studied the effects of group V and VII dopants on the electronic properties of 1T-ZrSe_2_ monolayer^22^. Xu Zhao et al. have calculated electronic and magnetic properties of 1T-HfS_2_ by doping transition-metal atoms in 2016^23^. The same group, have reported the structural, electronic and magnetic properties of 3d TM atom-doped 1T-HfSe_2_ monolayers in 2017^24^ and the study of the effect of vacancy defects on pristine and Cr-doped monolayer 1T-HfS_2_ conducted in 2018^25^. Ma Xu, et al. have studied the electronic and magnetic properties of Mn-doped monolayer 1T-HfS_2_^26^. The experimental and theoretical studies of Li et al. indicated that Fe-doped on 1T-SnS_2_ have high optoelectronics performance^17^. Y. Hao et al. have calculated Ni-doped in 1T-MoS_2_ monolayer to demonstrate that TM Ni doping can boost the hydrogen evolution reaction activity of 1T-MoS_2_^27^. M Kar et al. have studied the structural properties, energetic stabilities and magnetic properties of 1T-PtSe_2_ monolayer doped by different 3d, 4d and 5d TMs^28^.

The NiS_2_ monolayer can be considered as an excellent candidate for use in semiconductor devices similar to other monolayers^29,30^, but the absence of magnetism limits its applications in spintronic devices. Thus, by substituting atoms, we want to engineer the electronic and magnetic properties of the 1T-NiS_2_ monolayer. Despite all the studies on TMDs, the doping of the NiS_2_ monolayer have not reported yet. In this work, we figure out the effect of 3d TM, V-, Cr-, Mn-, Fe- and Co-doping on the structural, electronic and magnetic properties of 1T-NiS_2_ monolayer by using density functional theory (DFT)-based calculations. Our results show that V, Cr, Mn and Fe substitutions create magnetic pattern in 1T-NiS_2_ monolayer. The rest of the paper is as follow: we introduce the computational methods in “Computational methods” and we present the results in “Results and discussion”. The last section will be devoted to conclusion.

## Computational methods

We used DFT-based calculations to find the electrical and magnetic properties at 1T-NiS_2_ monolayer. The generalized gradient approximation (GGA) in the Perdew–Burke–Ernzerhof (PBE) form was used to represent the exchange–correlation functional of electrons. In all simulations, the kinetic energy of cutoff plane-waves is equal to 400 Ry. During structural optimization, the forces on each atom are smaller than 0.05 eV/Å under the periodic boundary condition. The Brillouin zone was sampled using a 4 × 4 × 1 Monkhorst–Pack k-point mesh for the 3 × 3 × 1 1T-NiS_2_ supercell^31^. The 1T-NiS_2_ monolayer is including 27 atoms (containing 9 Ni and 18 S atoms), in which a Ni atom is substituted by a Mn, Fe or Co atom in doping situation. The difference between valence electrons of substituted atoms induces new electrical and magnetic properties in the monolayer. The valence electrons for S, V, Cr, Mn, Fe, Co and Ni are 4s^2^3p^4^, 4s^2^3d^3^, 4s^1^3d^5^, 4s^2^3d^5^, 4s^2^3d^6^, 4s^2^3d^7^ and 4s^2^3d^8^, respectively.

## Results and discussion

### Electronic properties of pure 1T-NiS_2_ monolayer

**Figure 1:**
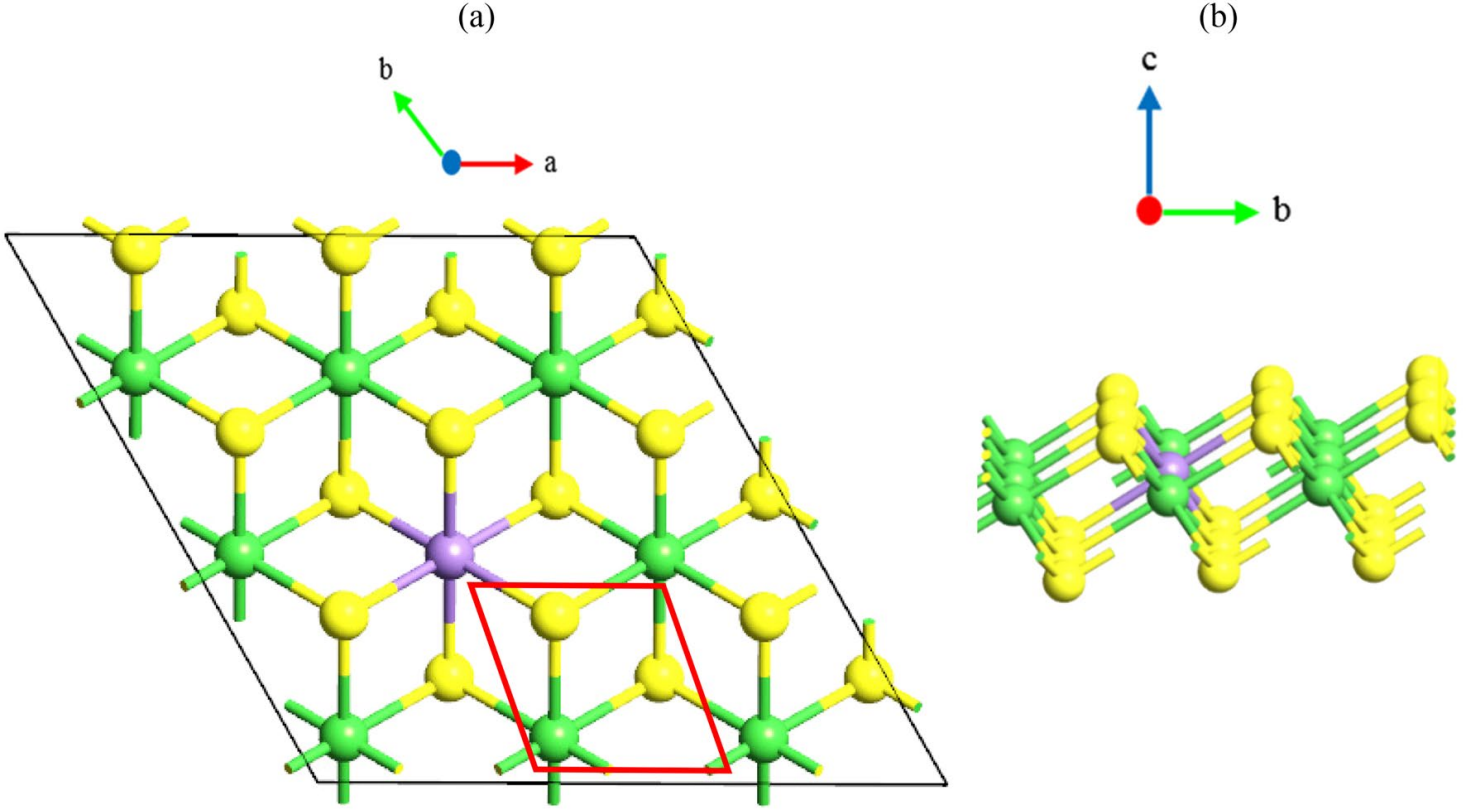
(**a**) Top and (**b**) side views of atomic structure for the doped 3 × 3 × 1 1T-NiS_2_ monolayer. Ni, S and TM atoms are represented by green, yellow and purple balls, respectively. The unit cell is denoted by the red frame.

**Figure 2:**
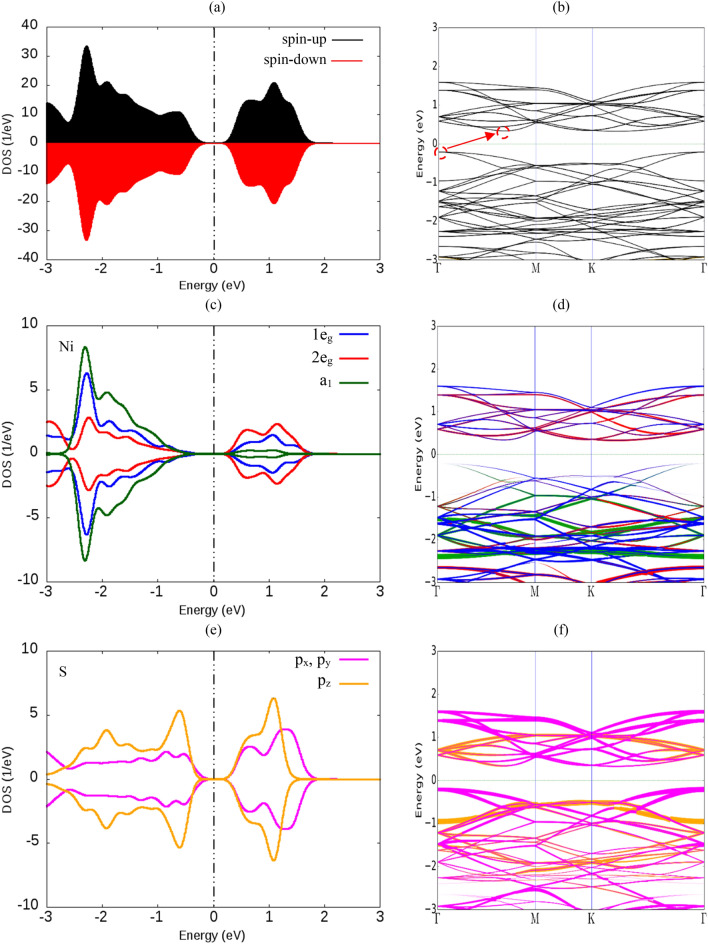
(**a**) TDOS and (**b**) band structure of 1T-NiS_2_ monolayer. (**c,d**) show the PDOS and band structure of the monolayer on d orbitals of Ni atoms, respectively. (**e,f**) denote to the contribution of p orbitals of S atoms on the DOS and band structure of the monolayer, respectively. The positive (negative) amounts of DOSs denote to spin-up (spin-down) states. The vertical dashed line represents the Fermi level which is set to zero.

The NiS_2_ monolayer has 1T and 2H phases and we checked both phases for stability. The results of this study showed that the total energy of 1T phase is 5.273 eV more than 2H phase, so 1T phase is more stable. In addition, 1T phase is a semiconductor, while 2H phase is a metal. Therefore, 1T phase of the NiS_2_ monolayer is considered to be doped by TM atoms. Because the band structure engineering is efficiently accessible in semiconductors. Also, to investigate the effects of substitution doping, we first study the structural, electronic and magnetic properties of the pure 1T-NiS_2_ monolayer. The pure 1T-NiS_2_ monolayer belongs to the hexagonal family with space group $\hbox{p}\overline{3}\hbox{m1}$. Figure 1 shows the top view (a) and side view (b) of a 3 × 3 × 1 supercell for doped 1T-NiS_2_ monolayer in which Ni atoms are in contact with six S atoms. Our calculations show that the lattice constant of the unit cell and the bond lengths between the Ni atom and the nearest S atoms are a = 3.35 Å and d_Ni-S_ = 2.26 Å, respectively, which is in agreement with the theoretical results reported recently^32^. Figure 2a,b shows the total density of state (TDOS) and the band structure of the pure 1T-NiS_2_ monolayer. It is clear that the monolayer is a non-magnetic semiconductor due to symmetric spin-up and spin-down DOSs. The valence band maximum is located at G k-point while the conduction band minimum is placed along G-M. The partial density of states (PDOSs) and band structure on d orbitals of Ni atoms and p orbitals of S atoms are shown in Fig. 2c–f. d orbitals of Ni atoms are split into two twofold degenerated 1e_g_ (d_xy_ and d_x_^2^_-y_^2^) and 2e_g_ (d_xz_ and d_yz_) and a single, a_1_ (d_z_^2^) states due to crystal field. a_1_ state has a more efficient contribution in the valence band, especially in the energy range of − 1 to − 2.5 eV than other d orbitals, but the same orbital has the lowest contribution in the conduction band. The conduction band originates mainly from 2e_g_ states in the energy range of 0.5 to 1.75 eV. Thus, the 1e_g_ state constitute the valence band maximum and the 2e_g_ state the conduction band minimum (see Fig. 2c,d). It is also clear that p_z_ orbital of S atoms has a significant contribution on the valence and conduction band in comparison with the p_x_ and p_y_ orbitals. the valence band maximum consists of the p_x_ and p_y_ orbitals and the conduction band minimum consists of the p_z_ orbital (see Fig. 2e,f). By considering the PDOSs, it reveals that the valence band maximum is consisted of p orbitals of S atoms, while the conduction band minimum is mainly made by the hybridization of d orbitals of Ni and p orbitals of S atoms. The exchange–correlation functional is an important term for investigation of band structure and band gap of 2D materials, so we have considered local density approximation (LDA), GGA, Heyd–Scuseria–Ernzerhof (HSE06) methods to investigate the band structure and band gap of 1T-NiS_2_ monolayer which is shown in Fig. S1 and S2 of the supplementary. The results show that the monolayer is an in-direct semiconductor regardless of the chosen method and the valence band maximum and the conduction band minimum location are approximately conserved. The obtained band gap by LDA and GGA is equal to 0.73 eV and 0.54 eV, respectively while it is 1.08 eV by HSE06. As it was predictable, the screened hybrid functional, HSE06, modified the band gap amount of the monolayer but it increases the cost of calculations. In many 2D materials studies, such as TM-doped in MoSe_2_^33^, HfS_2_^26,34^ and ZrS_2_^20^ monolayers, DFT calculations with GGA functional had been used and acceptable results had obtained, so we have reported the results calculated by GGA method for TM-doped monolayers. The spin–orbit coupling (SOC) has a considerable effect on the electronic structures such as band gap in semiconductor TMDs monolayers^35,36^. Therefore, we have investigated both GGA and GGA + SOC methods in the calculation of the band structure of 1T-NiS_2_ monolayer (see Fig. S1).

### Electronic and magnetic properties of 3d TM-doped 1T-NiS_2_ monolayer

For the doped systems, one Ni atom is substituted by one TM (TM = 3d group) atom in a 3 × 3 × 1 supercell. The calculated lattice constants (a), bond length between TM dopant and its first nearest neighbor S atom (d_TM-S_), total magnetic moments (μ_tot_), magnetic moments for the TM (μ_TM_) and its nearest neighbor Ni (μ_Ni_) and S (μ_S_) atoms and finally the energy difference between the spin-polarized and non-spin-polarized states (ΔE) of 1T-NiS_2_ monolayer are listed in Table 1. As can be seen in Table 1, the calculated total magnetic moments of V-, Cr-, Mn- and Fe-doped monolayers are 1.056 µ_B_, 2.651 µ_B_, 2.921 µ_B_ and 1.735 µ_B_, respectively. But the total magnetic moments of other TM-doped (Sc, Ti, Co, Cu, and Zn) 1T-NiS_2_ monolayers are zero. Therefore, these systems are non-magnetic and don't create any magnetization in the monolayer. In Ref.^21^, similar results reported for doped ZrSe_2_ monolayer. In addition, the non-magnetic behavior of the monolayers has been demonstrated by Sc and Ti doping in ZrS_2_^20^, HfS_2_^23^ and HfSe_2_^24^. Whereas, for V-, Cr-, Mn- and Fe-doped monolayer, the magnetic moment mainly locates on the V, Cr, Mn and Fe atoms (more details are given later). As is obvious from the ​​obtained values in Table 1, for V-doped, the V atom has local magnetic moment and total magnetic moment 1.029 μ_B_ and 1.056 μ_B_, respectively. But, a very small amount of magnetic moment distributes on its surrounding atoms. For V-doped, each of its six nearest neighbor Ni and S atoms show a magnetic moment 0.042 μ_B_ and − 0.057 μ_B_, respectively. On the other hand, for Cr-, Mn- and Fe-doped monolayers, Cr, Mn and Fe atoms have local magnetic moments 2.639 μ_B_, 2.913 μ_B_ and 1.726 μ_B_ and total magnetic moment 2.651 μ_B_, 2.921 μ_B_ and 1.735 μ_B_, respectively. In Cr-, Mn- and Fe-doped, like V-doped, surrounding atoms have a very small amount of magnetic moment. According to obtained results, coupling between TM atoms (V, Cr, Mn and Fe) and S atoms is anti-ferromagnetic like coupling between Ni atoms and Fe atoms in Fe-doped monolayer, while coupling between Ni atoms with V, Cr and Mn atoms for V-, Cr- and Mn-doped monolayers is ferromagnetic. Also, the obtained results in Table 1 show that the bond lengths between TM and S atoms are reduced from Sc to Co by the reduction of atomic radius from Sc to Co and then this value increases for Cu and Zn with increasing atomic radius for Cu and Zn. In addition, the bond length of Co-S in the Ni_8_CoS_18_ system is shorter than other doped systems, which suggesting the stronger covalent interaction. In following, we calculate the energy differences between the spin-polarized and non-spin-polarized, i.e., ∆E = E_sp_-E_nsp_ for TM-doped 1T-NiS_2_ monolayers. We can see that the V-, Ti-, Mn- and Fe-doped 1T-NiS_2_ monolayers display the negative energy differences ∆E, indicating the spin-polarized state is more stable than the non-spin-polarized state. By comparing the energy differences, it is observed that Mn-doped than V-, Cr- and Fe-doped has higher energy differences and spin-polarized pattern, while the energy differences between the spin-polarized and non-spin-polarized for other TM-doped monolayers is zero. So we illustrated the details of obtained results for Mn- and Fe-doped as a magnetic semiconductor and a half-metal and Co-doped monolayer as a representative of a non-magnetic system in the main text. To find a comprehensive view, the band structure, DOS, spin density and local magnetic moments of V- and Cr-doped 1T-NiS_2_ monolayers are given in Fig. S3, S5 and S6, respectively, of the supplementary.

**Table 1:** The lattice constants (a), the bond length between doped TM and the nearest S atoms (d_TM-S_), the total magnetic moments (μ_tot_), the magnetic moments for the dopant (μ_TM_) and its nearest neighbor Ni (μ_Ni_) and S (μ_S_) atoms and the energy difference between the spin-polarized and non-spin-polarized states (ΔE).

	a (Å)	d_TM-S_ (Å)	µ_tot_ (µ_B_)	µ_TM_ (µ_B_)	µ_Ni_ (µ_B_)	µ_S_ (µ_B_)	ΔE (meV)
Ni_8_ScS_18_	9.984	2.558	0	0	0	0	0
Ni_8_TiS_18_	9.981	2.438	0	0	0	0	0
Ni_8_VS_18_	9.982	2.361	1.056	1.029	0.042	− 0.057	− 62.73
Ni_8_CrS_18_	9.972	2.320	2.651	2.639	0.045	− 0.087	− 661.49
Ni_8_MnS_18_	9.973	2.285	2.921	2.913	0.015	− 0.052	− 1027.81
Ni_8_FeS_18_	9.961	2.258	1.735	1.726	− 0.019	− 0.002	− 247.68
Ni_8_CoS_18_	9.987	2.254	0	0	0	0	0
Ni_8_CuS_18_	10.061	2.353	0	0	0	0	0
Ni_8_ZnS_18_	9.990	2.497	0	0	0	0	0

Finally, to understand the stability of the desired monolayers, the binding energy is calculated by E_b_ = [E_Ni8TMS18_ − E_TM_ − 8E_Ni_ − 18E_S_]/27. The binding energy of the pure monolayer is equal to − 4.176 eV/atom. On the other hand, the binding energy for V-, Cr-, Mn-, Fe- and Co-doped monolayers is − 4.237, − 4.149, − 4.147, − 4.178 and − 4.178 eV/atom, respectively. According to the calculated values, the binding energy of the V-, Fe- and Co-doped are less than pure, Cr- and Mn-doped monolayers. So, V-, Fe- and Co-doped monolayers are considered as the most stable states. In fact, the structure is more inclined to absorb V, Fe and Co atoms.

**Figure 3:**
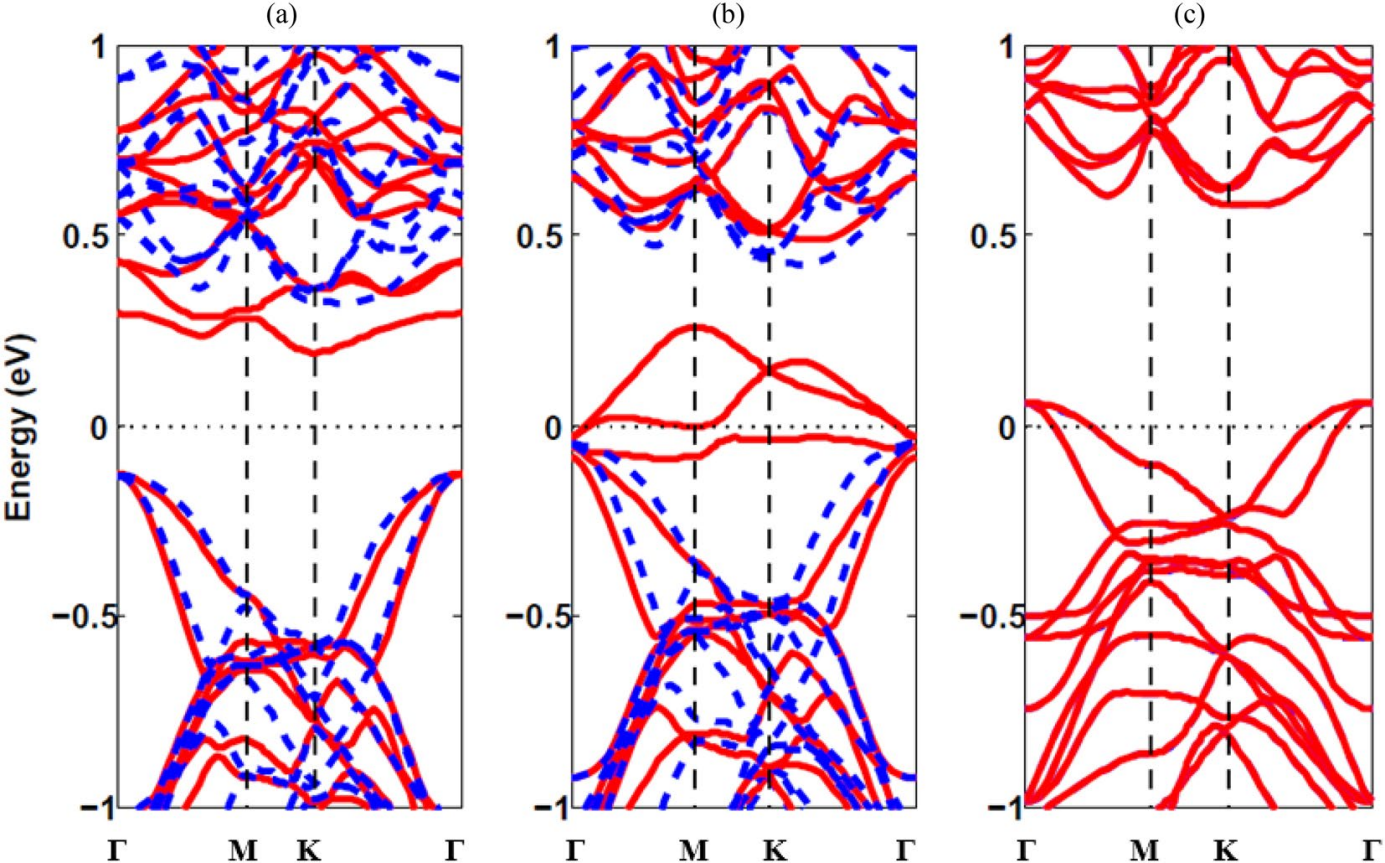
Band structures of (**a**) Mn-doped, (**b**) Fe-doped and (**c**) Co-doped 1T-NiS_2_ monolayer. The blue and red lines represent the spin-up and spin-down, respectively. Fermi level is located in zero energy.

In order to study the effect of doping on electronic and magnetic properties, we calculate the band structures 1T-NiS_2_ monolayer in the presence TM atoms and the results are shown in Fig. 3. Depending on the species of TM atoms, the substituted 1T-NiS_2_ monolayer can be a semiconductor, half-metal or metal. Figure 3a shows that in the Mn-doped monolayer, the spin-up and spin-down channels are asymmetric. So, this system is a magnetic semiconductor. In fact, the presence of Mn atom in the monolayer causes spin separation and changes the bandgaps. On the other word, when the Ni atom is replaced by Mn atom, the bands dispersion is changed in both majority spin and minority spin bands and the spin-splitting has occurred. Similarly, Mn-doped HfS_2_^34^, HfSe_2_^24^ and ZrSe_2_^37^ monolayers are magnetic semiconductor. In the case of Fe-doped (V-doped), as shown in Fig. 3b (see Fig. S3 (a)), several spin-down (spin-up) branches cross the Fermi level, while the spin-up (spin-down) band structures remain semiconductor. Therefore, this system is a half-metal, like Fe-doped in SnS_2_ monolayer^38^ (V-doped in HfSe_2_ monolayer^24^). Half-metal systems are generally the source of quite spin-polarized electrons which are promising for development of high performance spintronic devices. However, from Fig. 3c (Fig. S3 (b)), we can see that in the Co-doped (Cr-doped) case, the symmetric (asymmetric) spin-up and spin-down channels pass through the Fermi level, showing a non-magnetic (magnetic) metallic behavior, which is similar to the behavior of Co-doped HfSe_2_ monolayer^24^ (Cr-doped HfS_2_^23^ and HfSe_2_^24^ monolayers). On the other hand, we know that the valence electrons of Co atoms are in 3d^7^4s^2^ configuration. So p-type doping is created in Co-doped monolayer because the substituted Co atom has one electron less than Ni. Due to the presence of massive TM atoms in the monolayers, the effect of SOC should be noticed. The band structure of TM-doped monolayers with SOC are given in Fig. S4 of the supplementary. It shows that the amount of band gap is slightly reduced with SOC calculations.

**Figure 4:**
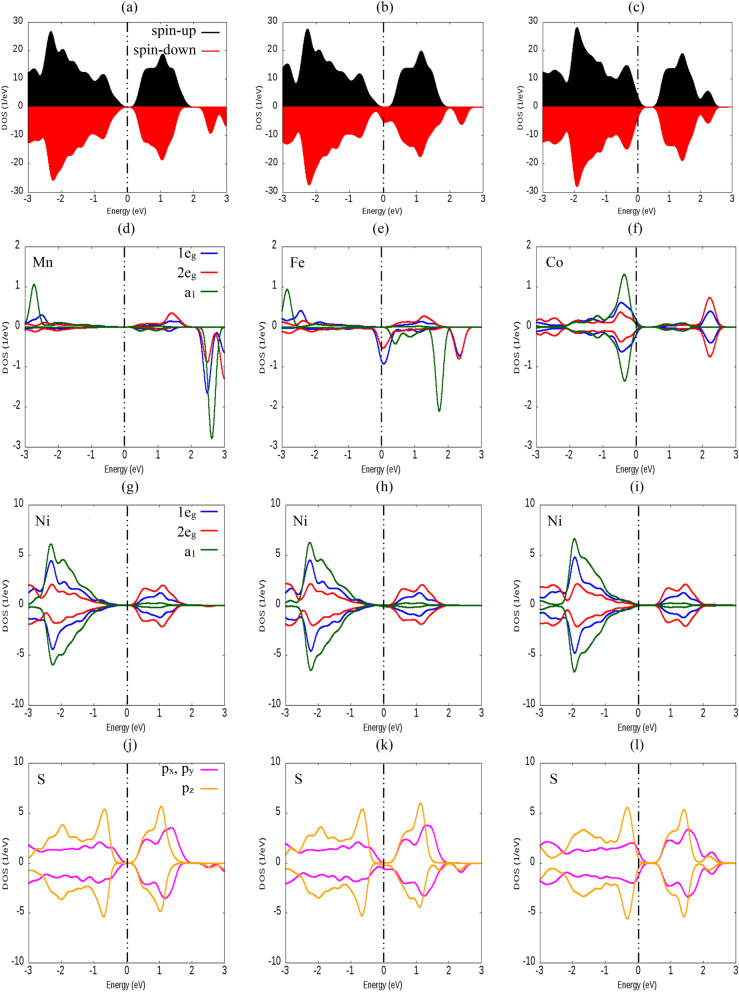
(**a–c**) show TDOS of Mn-, Fe- and Co-doped 1T-NiS_2_ monolayers, respectively. (**d–f**) denote to the projection of the monolayer DOS on d orbitals of doped atoms, while (**g–i**) refer to the projection of the monolayer DOS on d orbitals of Ni atoms and (**j–l**) represent the contribution of p orbitals of S atoms on the DOS of Mn-, Fe-, Co-doped monolayers, respectively. The vertical dashed line represents the Fermi level which is set to zero.

**Figure 5:**
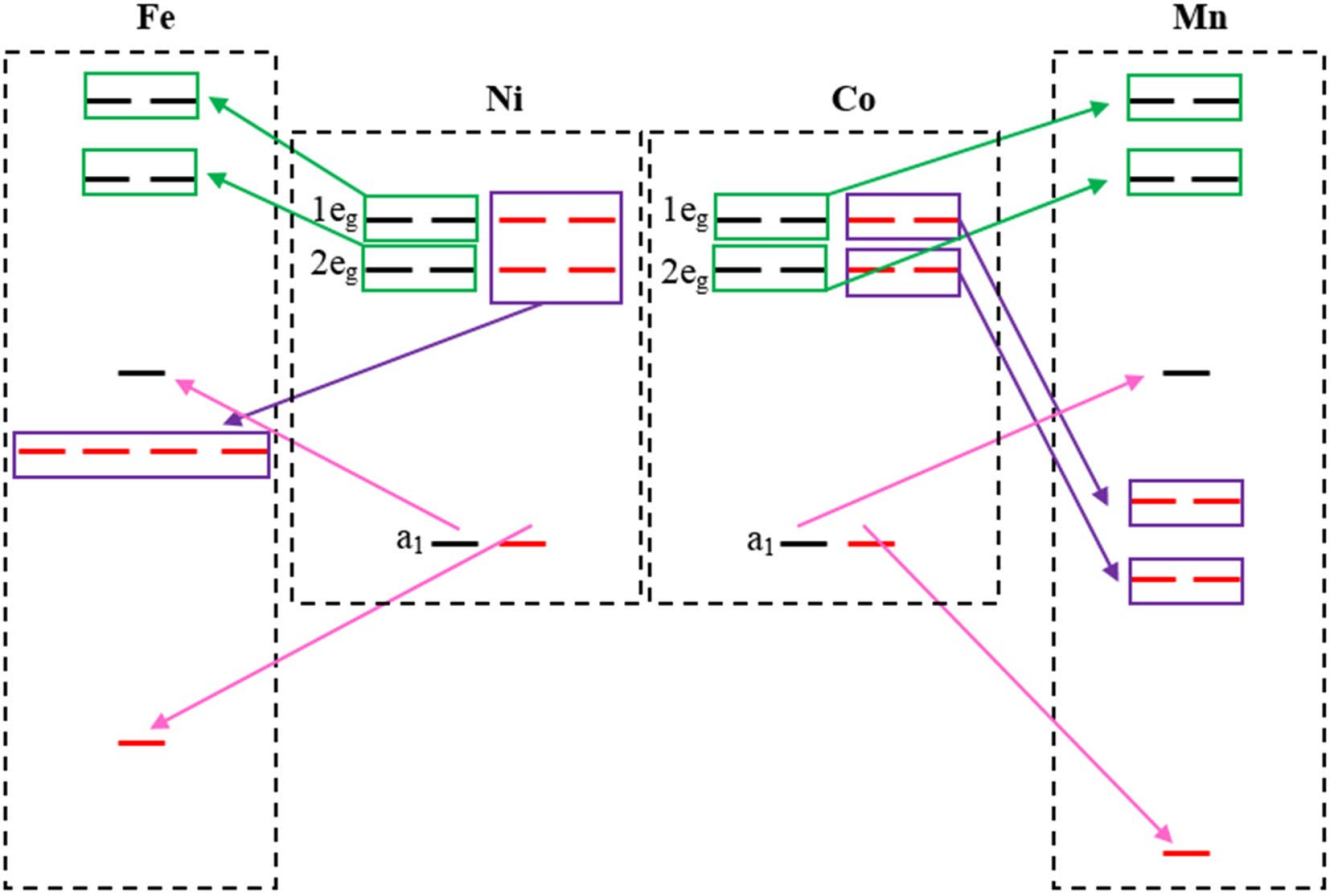
Schematic energy level diagram of TM-doped NiS_2_ monolayer.

The TDOSs and PDOSs onto p orbitals of S, d orbitals of substituted TM and Ni atoms are displayed in Fig. 4. Figure 4a–c displays TDOS for spin-up and spin-down of Mn-, Fe- and Co-doped monolayers. Also, 1e_g_, 2e_g_ and a_1_ states of TM atoms (Mn, Fe, Co and Ni) and p_x_, p_y_ and p_z_ orbitals of chalcogen atoms (S) are drawn separately in Fig. 4d–l. The energy and splitting of these states vary significantly depending on the kind of doped TM atoms. TDOS and PDOS for V- and Cr-doped monolayers are showed in Fig. S5 of the supplementary. In the case of Mn-doped, the asymmetric TDOS is occurred for spin-up and spin-down surrounding the Fermi level. So, as we mentioned earlier this system is a magnetic semiconductor with total magnetic moment of 2.921 μ_B_ (see Table 1). For Fe-doped 1T-NiS_2_ monolayer, in the spin-down channel, the doping states pass through the Fermi level showing a metallicity, which are derived mostly from the p_x_ and p_y_ orbitals of S atom with significant contribution of 1e_g_ and 2e_g_ states of Fe atom. Whereas, the spin-up channel remains semiconductor, which are derived mostly of p_x_ and p_y_ orbitals of S atoms in the valence band maximum and also the significant contribution of 2e_g_ state of Fe atom and p_z_ orbitals of S atoms are observed in the conduction band minimum. As a result, Fe-doped monolayer with μ_tot_ = 1.735 μ_B_ (see Table 1) is a half-metal. Thus, Fe-doped monolayer can be used in spintronic material operating as a spin valve or can be an active component in 2D magnetic tunnel junctions. Finally, Co-doped in 1T-NiS_2_ monolayer display a metallic behavior for both spin-up and spin-down bands that crossing the Fermi level. In addition, the spin-up and spin-down channels are symmetric, indicating the Co-doped 1T-NiS_2_ monolayer is a non-magnetic metal, hence as shown in Table 1, Ni_8_CoS_18_ system has no magnetic moment. In fact, in this system, p orbitals of S and d orbitals of Co atoms are mainly responsible for the metallicity of the Co-doped 1T-NiS_2_ monolayer. To understand the magnetic moment according to PDOS of the monolayers, it is observed that both spin-up and spin-down, degenerated 1e_g_ and 2e_g_ and single a_1_ states of d orbital are below the Fermi level and hence fully occupied in Co-doped monolayer (see Fig. 4f). Therefore, its magnetic moment becomes zero. In Mn- and Fe-doped monolayers, it is clear that only spin-up single a_1_ state is located below the Fermi level, so it is occupied. But spin-down single a_1_ state is located at top of the Fermi level which is empty. For this reason, they have non-zero magnetic moments. According to the Fig. 4f, it is obvious that no exchange splitting exists in the d orbitals of Co atom, while the exchange splitting is happened for d orbitals of Mn and Fe atoms (see Fig. 4d,e). A schematic graph of the spin states of TM-d orbitals is represented in Fig. 5. The states of Co atom are degenerated and there is no spin separation. For Mn and Fe atoms, the spin separation is quite noticeable. On the other hand, the degeneration of spin-down 1e_g_ and 2e_g_ states of Fe atom at zero energy (see Fig. 4e) makes a half-metal Fe-doped monolayer.

**Figure 6:**
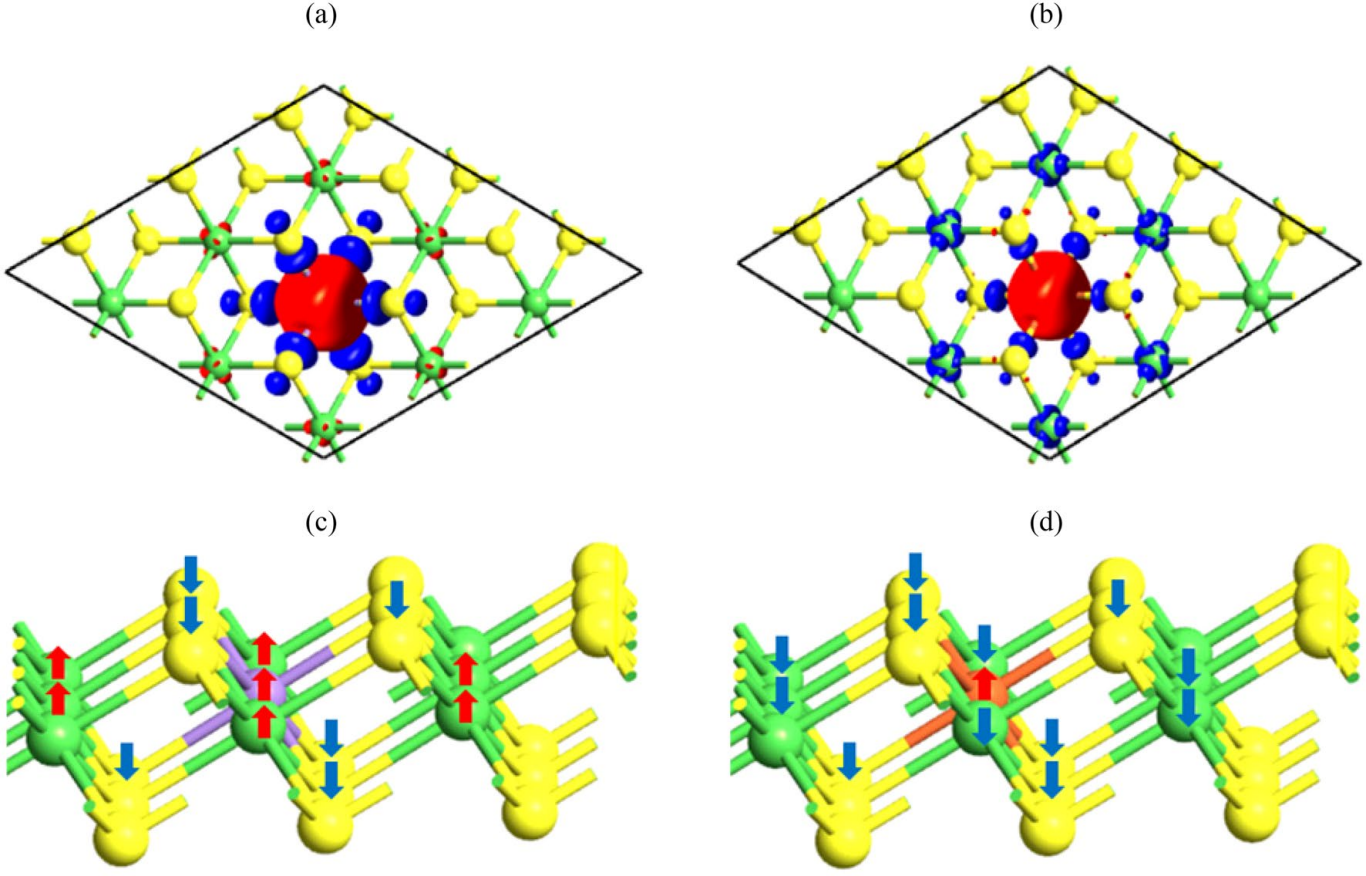
The spin density (ρ_↑_–ρ_↓_) (first row) and the local magnetic moment (second row) plots for Mn-doped (**a,c**) and Fe-doped (**b,d**) 1T-NiS_2_ monolayers. The red (blue) isosurfaces represent positive (negative) spin densities of 0.02 e Å^-3^. Also, the blue and red arrows in local magnetic moments are drawn to indicate the orientation of spin polarization of the TM (Mn, Fe and Ni) and chalcogen (S) atoms.

As correct evidence of the charge transfers between the doped atoms and 1T-NiS_2_ monolayer, we plot the spin density (ρ_↑_–ρ_↓_) for Mn- and Fe-doped systems in Fig. 6a,b. Also, the spin density for V- and Cr-doped monolayers are showed in Fig. S6a,b of the supplementary. ρ_↑_ and ρ_↓_ show the charge density of spin-up and spin down states, respectively. From the magnetic moment of the TM and its nearest neighbor Ni/S, as listed in Table 1, we found that the significant contribution of the total magnetic moment comes from the TM atom. These results are in agreement with the spin density as shown in Fig. 6. In the case of the Mn-doped monolayer (Fig. 6a), the positive magnetic moment of the Mn atom induces the negative (positive) magnetic moments on the first nearest-neighbor S (Ni) atoms (see Table 1). In addition, Fig. 6a shows that the Mn atom induces magnetic moments only on the first nearest-neighbor S atoms and this amount is greater than the induced positive magnetic moments on the first nearest-neighbor Ni atoms. On the other hand, the S atoms are easier to be magnetized rather than Ni atoms in Mn-doped monolayer. So, coupling between Mn atom and S (Ni) atoms is anti-ferromagnetic (ferromagnetic), like V- and Cr-doped monolayers (see Fig. S6a,b). Unlike V-, Cr- and Mn-doped monolayers, in the Fe-doped monolayer, the positive magnetic moment of the Fe atom induces the negative magnetic moments on the first nearest-neighbor S and Ni atoms. According to Fig. 6b, it is easier for Ni atoms to be magnetized rather than S atoms in Fe-doped monolayer. Thus, Fe and S/Ni atoms are coupled anti-ferromagnetically. The alignments of local magnetic moments (µ_TM_ and µ_S_) for Mn- and Fe-doped monolayers are shown in Fig. 6c,d. Also, the alignments of local magnetic moments for V- and Cr-doped monolayers are displayed in Fig. S6c,d. In the V-, Cr- and Mn-doped monolayers, the magnetic moment of the V-, Cr- and Mn atoms and the first nearest-neighbor Ni (S) atoms are parallel (anti-parallel). For example, as shown in Table 1, Ni_8_MnS_18_ system provided a positive magnetic moment for Mn and Ni atoms, while it provided negative magnetic moment for S atoms which is clearly displayed in Fig. 6a,c. Whereas, the conditions for Fe-doped monolayer is completely different. In the Fe-doped monolayer, the magnetic moment of the Fe atom with the first nearest-neighbor Ni and S atoms are anti-parallel i.e., Ni_8_FeS_18_ system provided a positive magnetic moment for Fe atom, while provided negative magnetic moments for Ni and S atoms. It seems that in general the strong exchange interaction in Mn-doped monolayer causes the relatively small spin orientation of Ni atoms by Mn atom. While for Fe-doped monolayer, due to the weak exchange interaction, the equal orientation of the spins of the Ni atoms by Fe atom is not possible.

## Conclusions

In summary, we have investigated the effect of 3d transition metal, V-, Cr-, Mn-, Fe- and Co-doping on the structural, electronic and magnetic properties of 1T-NiS_2_ monolayer by using density functional theory calculations. The results show that pure 1T-NiS_2_ monolayer is a non-magnetic semiconductor. Whereas, depending on the species of substituted transition metal atom, this monolayer can be a magnetic semiconductor (Mn-doped), half-metal (V- and Fe-doped) and magnetic (Cr-doped) or non-magnetic (Co-doped) metal. The doped monolayers can be used in nanoelectronics and spintronics devices due to their controllable features. Moreover, the V-, Cr-, Mn- and Fe-doped induce total magnetic moments of 1.056 μ_B_, 2.651 μ_B_, 2.921 μ_B_ and 1.735 μ_B_, respectively, while Co atom does not induce any magnetic moment. These results indicate that the magnetism can be controlled by doping of 3d transition metal atoms. We hope that our result can be useful in the field of new 2D magnetic materials.

## Supplementary Information

Supplementary Figures.
