# Supplementary material for: Band structure engineering of NiS2 monolayer by transition metal doping
Source: Sci Rep. 2021 Mar 11;11:5779. doi: 10.1038/s41598-021-84967-3 (PMC7952594; doi:10.1038/s41598-021-84967-3)
Supplement: Supplementary file 1 — Supplementary Figures. [file 41598_2021_84967_MOESM1_ESM.docx]

**Band structure engineering of NiS_2_ monolayer by transition metal doping**

**H. Khalatbari^1^, S. Izadi Vishkayi ^2^, M. Oskouian^1^,**

**and H. Rahimpour Soleimani^1^**
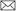


^1^Computational Nanophysics Laboratory (CNL), Department of Physics, University of Guilan, P. O. Box 41335-1914, Rasht, Iran.
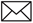
Email: Rahimpour@guilan.ac.ir

^2^School of Physics, Institute for research in fundamental science (IPM), P. O. Box 19395-5531, Tehran, Iran

| 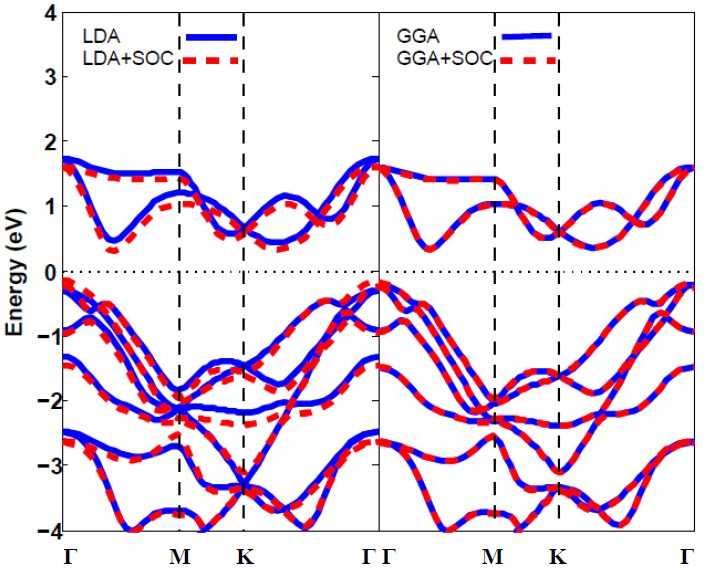 |
| --- |

**Figure S1.** The band structure of 1T-NiS_2_ unit cell by LDA and GGA exchange-correlation functional. The SOC is considered in the dashed lines. The calculated band gap by LDA, LDA+SOC, GGA and GGA+SOC methods is equal to 0.73 eV, 0.43 eV, 0.54 eV and 0.48 eV, respectively.

| 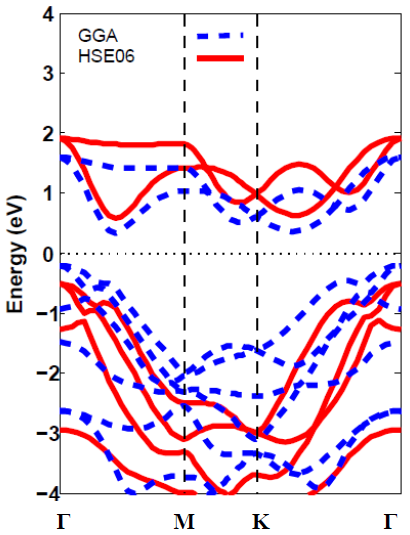 |
| --- |

**Figure S2.** The band structure of 1T-NiS_2_ unit cell by GGA and HSE06 exchange-correlation functional.

| (a) (b)  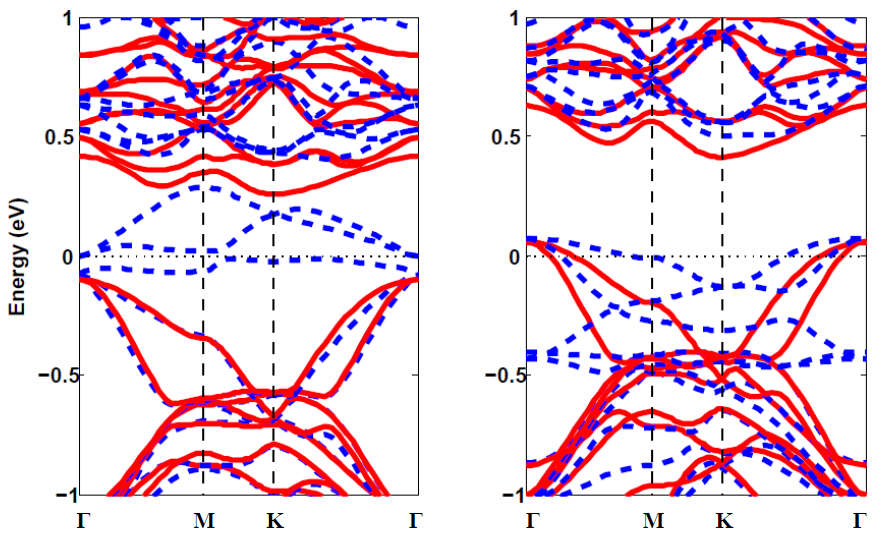 |
| --- |

**Figure S3.** Band structures of (**a**) V-doped and (**b**) Cr-doped 1T-NiS_2_ monolayer. The blue and red lines represent the spin-up and spin-down, respectively. Fermi level is located at zero energy.

| (a)  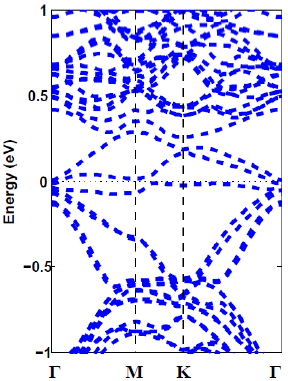 | (b)  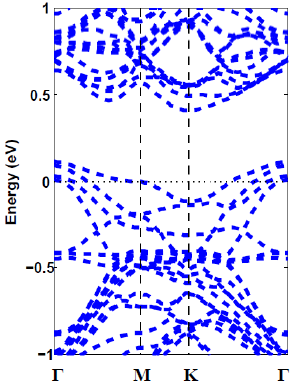 | | (c)  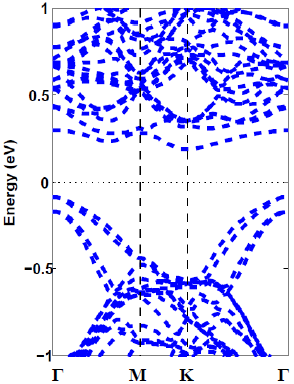 |
| --- | --- | --- | --- |
| (d)  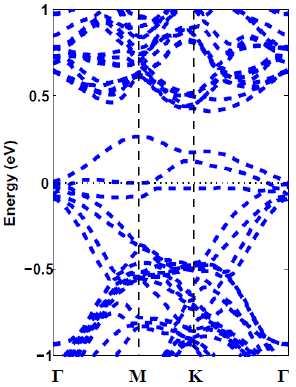 | | (e)  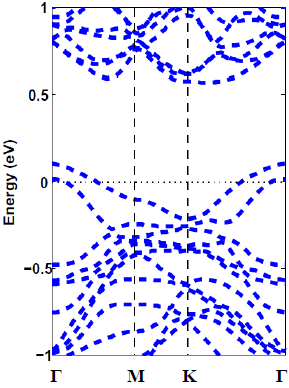 | |

**Figure S4.** The band structures of TM atoms ((**a**) V, (**b**) Cr, (**c**) Mn, (**d**) Fe and (**e**) Co) doped 1T-NiS_2_ monolayer with SOC calculation. Fermi level is located at zero energy.

| (a)  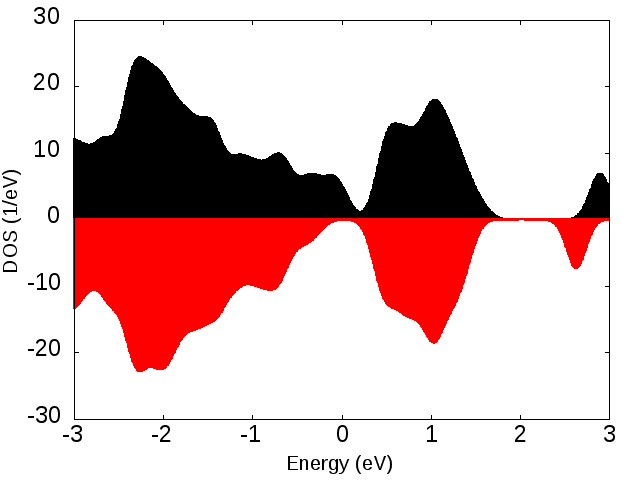  spin-up  spin-down | (b)  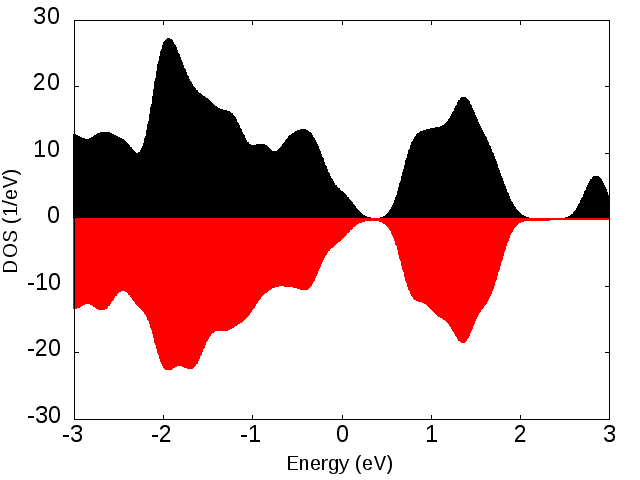 |
| --- | --- |
| (c)  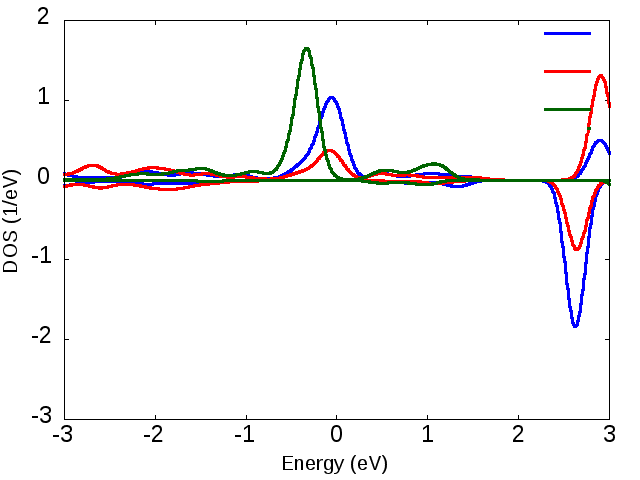  V  1e_g_  2e_g_  a_1_ | (d)  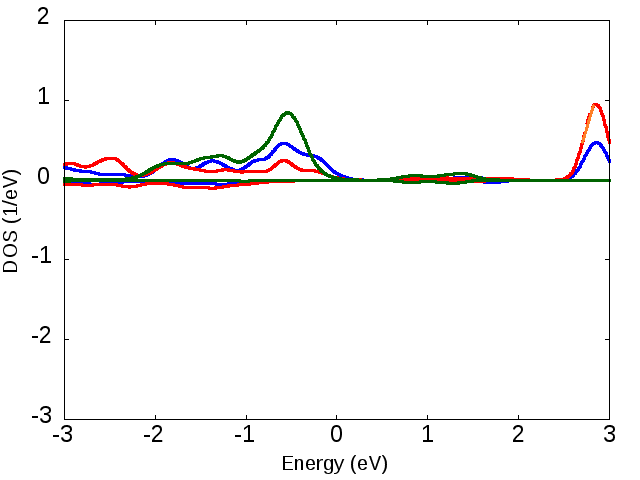  Cr |
| (e)  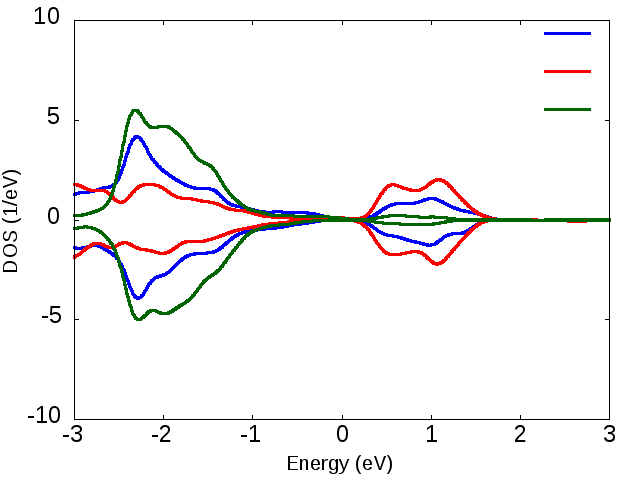  Ni  1e_g_  2e_g_  a_1_ | (f)  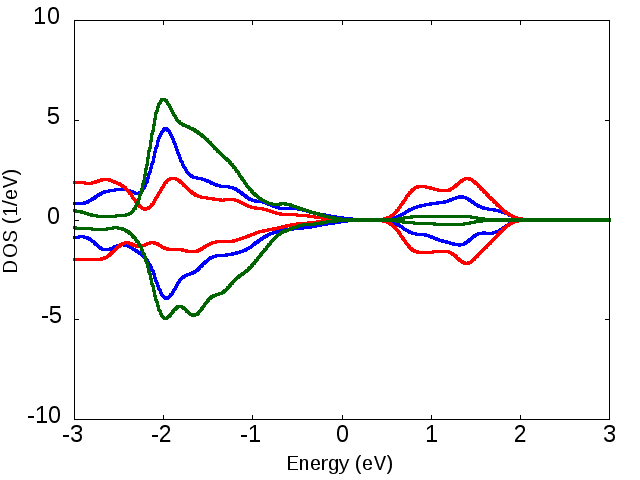  Ni |
| (g)  p_x_, p_y_  p_z_  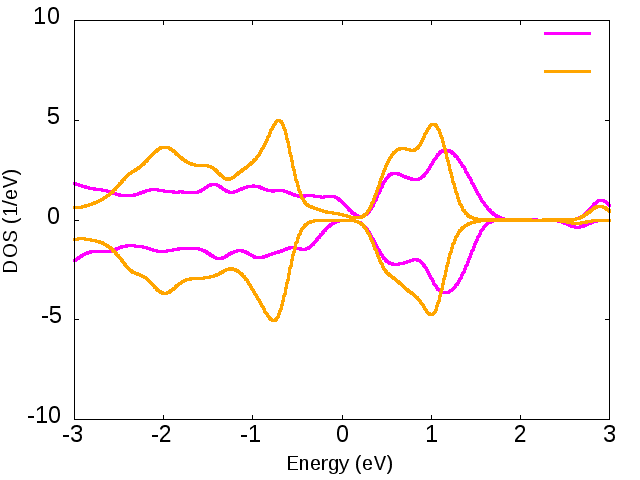  S | (h)  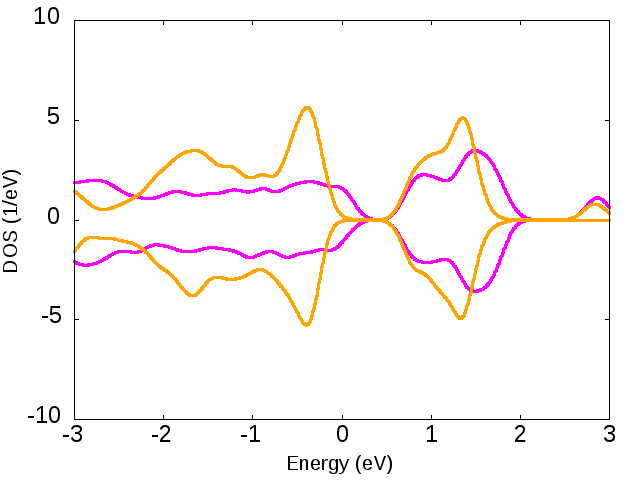  S |

**Figure S5.** (**a**) and (**b**) show TDOS of V- and Cr-doped 1T-NiS_2_ monolayers, respectively. (**c**) and (**d**) denote to the projection of the monolayer DOS on d orbitals of doped atoms, while (**e**) and (**f**) refer to the projection of the monolayer DOS on d orbitals of Ni atoms and (**g**) and (**h**) represent the contribution of p orbitals of S atoms on the DOS of V-, Cr-doped monolayers, respectively. The vertical dashed line represents the Fermi level which is set to zero.

| (a)   | (b)   |
| --- | --- |
| (c)   | (d)   |

**Figure S6.** The spin density (ρ_↑_-ρ_↓_) (first row) and the local magnetic moment (second row) plots for V-doped ((**a**) and (**c**)) and Cr-doped ((**b**) and (**d**)) 1T-NiS_2_ monolayers. The red (blue) isosurfaces represent positive (negative) spin densities of 0.02 e Å^-3^. Also, the blue and red arrows in local magnetic moments are drawn to indicate the orientation of spin polarization of the TM (V, Cr and Ni) and chalcogen (S) atoms.
